# Supplementary figures and images for: Intellectual disability-associated disruption of O-GlcNAc cycling impairs habituation learning in Drosophila
Source: PLoS Genet. 2022 May 2;18(5):e1010159. doi: 10.1371/journal.pgen.1010159 (PMC9140282; doi:10.1371/journal.pgen.1010159)

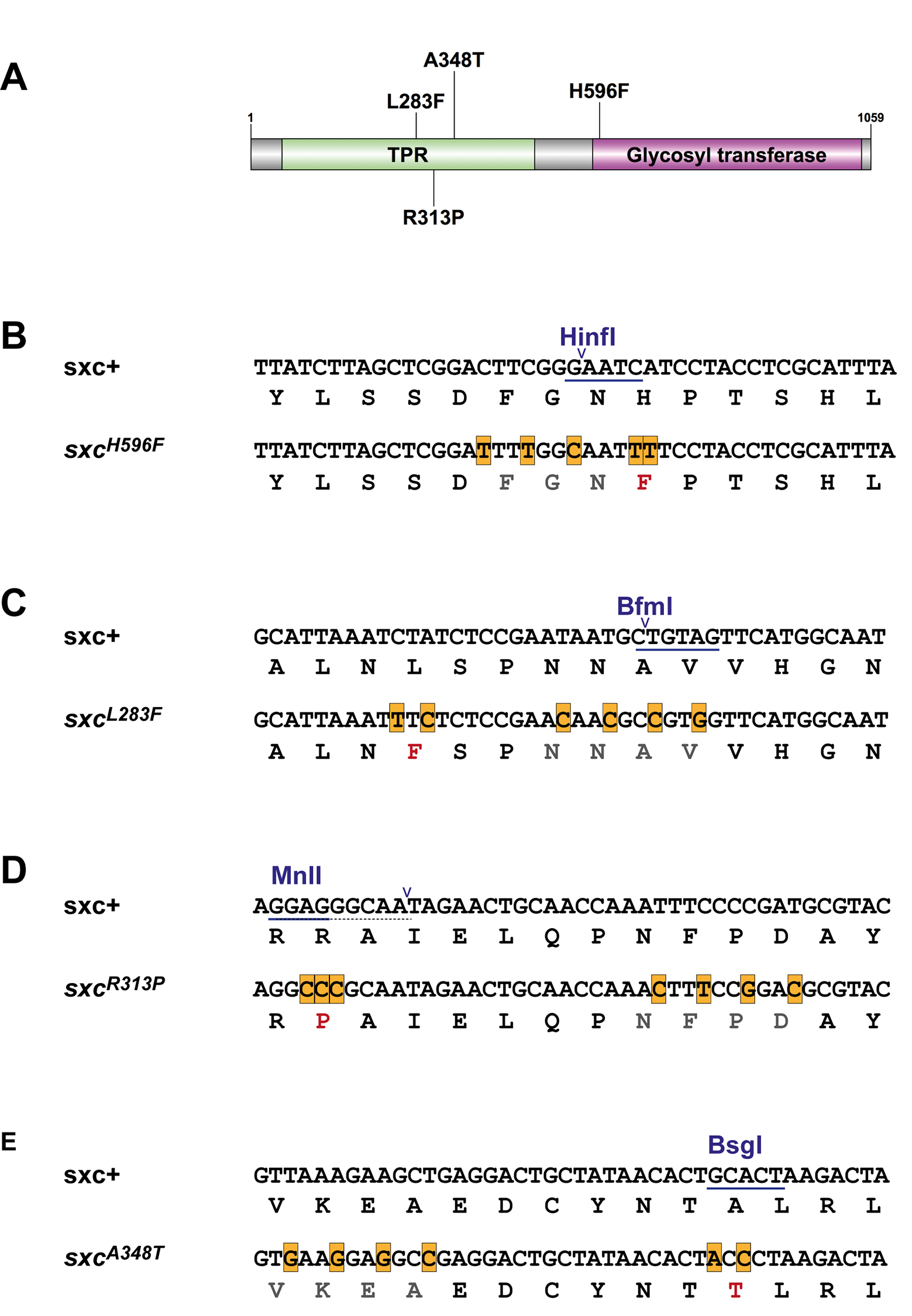

Supplement: S1 Fig — (A) Schematic representation of Drosophila sxc protein showing the location of H596F, L283F, R313P and A348T mutations; purple tetratricopeptide repeat (TPR) domain, green glycosyl transferase (GT) domain. (B)–(E) Sequences of genomic DNA of wild type, sxcH596F, sxcL283F, sxcR313P and sxcA348T Drosophila alleles. The missense mutation and additional silent mutations are highlighted. The restriction digestion sites used for genotyping are shown. (TIF) [file pgen.1010159.s001.tif]

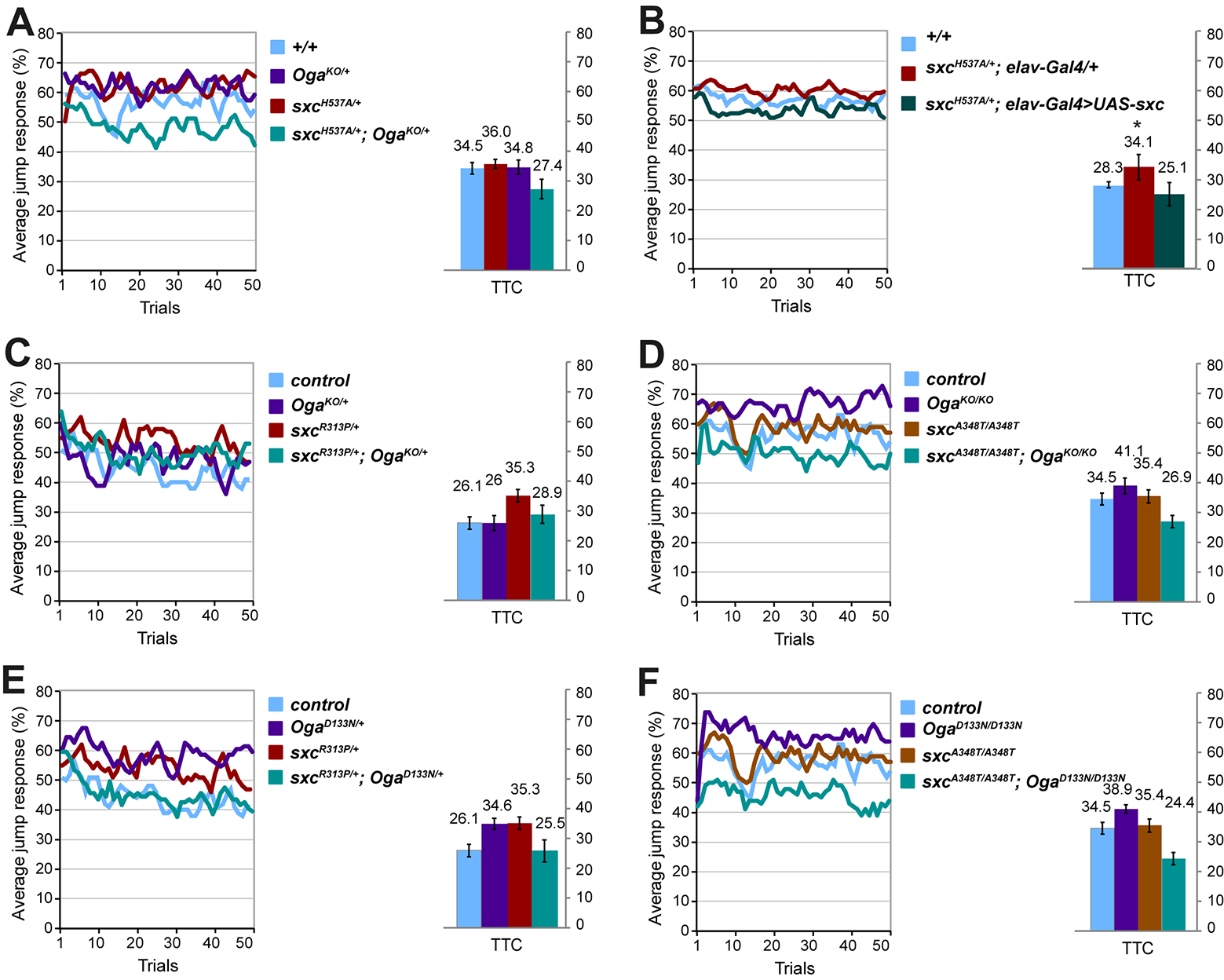

Supplement: S2 Fig — In the fatigue assay, jump responses were induced with 50 light-off pulses with 5 s interval between pulses that prevents habituation. The jump response is presented as % of jumping flies in each light-off trial. The mean number of trials that flies needed to reach the no-jump criterion (Trials To Criterion, TTC) ± SEM is presented. (A) Jump response of the sxcH537A/+; OgaKO/+ flies (N = 85, mean TTC ± SD: 27.4 ± 8, in cyan) remains high throughout the entire course of the experiment, similar to control flies (+/+, N = 85, mean TTC ± SD: 34.5 ± 5, padj = 0.14, in blue) demonstrating that restored habituation in sxcH537A/+; OgaKO/+ flies (Fig 1B) is not confounded by fatigue. (B) Jump response of the sxcH537A/+; elav-Gal4>UAS-sxc flies (N = 52, mean TTC ± SD: 25.1 ± 7.9, in green) remains high throughout the entire course of the experiment, similar to control flies (+/+, N = 55, mean TTC ± SD: 28.3 ± 2.3, padj = 0.128, in blue) demonstrating that restored habituation in sxcH537A/+; elav-Gal4>UAS-sxc flies (Fig 1D) is not confounded by fatigue. (C) Jump response of the sxcR313P/+; OgaKO/+ flies (N = 73, mean TTC ± SD: 28.9 ± 7.6, in cyan) remains high throughout the entire course of the experiment, similar to control flies (+/+, N = 84, mean TTC ± SD: 26.1 ± 5.1, padj = 1, in blue) demonstrating that restored habituation in sxcR313P/+; OgaKO/+ flies (Fig 4C) is not confounded by fatigue. (D) Jump response of the sxcA348T/A348T; OgaKO/KO flies (N = 78, mean TTC ± SD: 26.9 ± 5.1, in cyan) remains high throughout the entire course of the experiment, similar to control flies (+/+, N = 85, mean TTC ± SD: 34.5 ± 5, padj = 0.31, in blue) demonstrating that restored habituation in sxcA348T/A348T; OgaKO/KO flies (Fig 4D) is not confounded by fatigue. (E) Jump response of the sxcR313P/+; OgaD133N/+ flies (N = 83, mean TTC ± SD: 25.5 ± 9.2, in cyan) remains high throughout the entire course of the experiment, similar to control flies (+/+, N = 84, mean TTC ± SD: 26.1 ± 5.1, padj [file pgen.1010159.s002.tif]

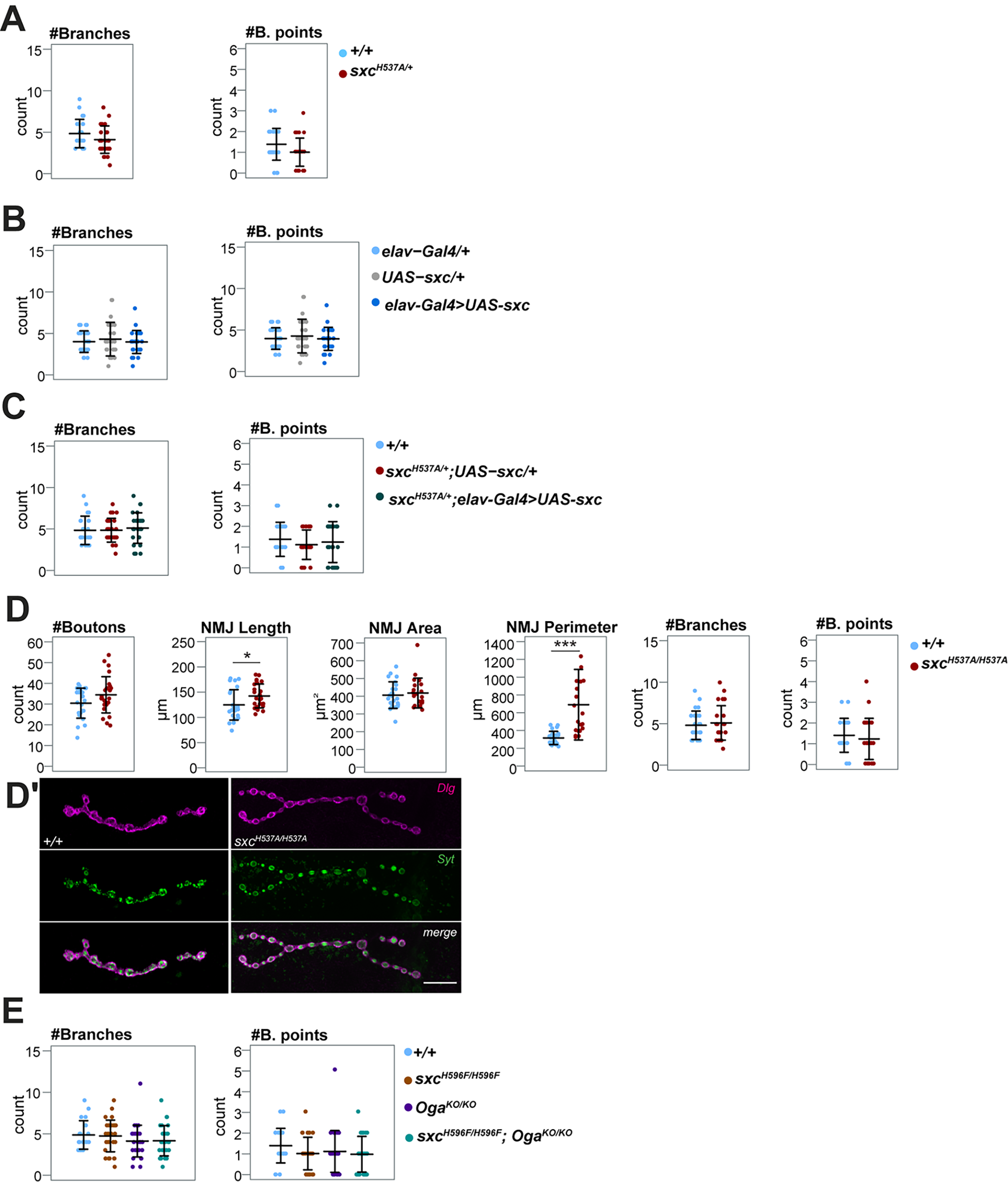

Supplement: S3 Fig — (A) Number of synaptic branches and branching points in sxcH537A/+ larvae (N = 29, in red) is not significantly different from the genetic background control larvae (+/+, N = 24, branches: p = 0.1439, branching points: p = 0.05648, in blue). P-values are based non-parametric Wilcoxon test analysis. (B) Number of branches and branching points is not affected in elav-Gal4>UAS-sxc larvae (N = 29, in dark blue) compared the elav-Gal4/+ larvae (N = 30, branches: padj = 0.8702, branching points: padj = 0.8488, in light blue) and to UAS-sxc/+ larvae (N = 26, branches: padj = 0.8294, branching points: padj = 0.2689, in grey). (C) Branches and branching points are not affected in sxcH537A/+; UAS-sxc/+ larvae (N = 28, in red) compared to the control larvae (+/+, N = 28, branches: padj = 0.7121, branching points: padj = 0.2979, in blue). sxcH537A/+; elav-Gal4>UAS-sxc larvae (N = 29, in green) do not show any changes in number of branches and branching points compared to the sxcH537A/+; UAS-sxc/+ larvae (branches: padj = 0.4097, branching points: padj = 0.5928) and control larvae (+/+; branches: padj = 0.4301, branching points: padj = 0.6927). (D) sxcH537A/H537A larvae have significantly increased NMJ length (N = 25, p =) and perimeter (N = 21, p =, in red) compared to their genetic background control (+/+, N = 26, in blue) but not significantly different number of boutons (p = 0.085), NMJ area (p = 0.618), number of branches (p = 0.691) and branching points (p = 0.371). * p<0.05, *** p<0.001. P-values for boutons, length, area and perimeter are based on one-way ANOVA. P-values for branches and branching points are based on non-parametric Wilcoxon test analysis. (E) Branches and branching points are not affected in sxcH956F larvae (N = 31, branches: padj = 1, branching points: padj = 0.5, in brown), OgaKO larvae (N = 30, branches: padj = 0.75, branching points: padj = 0.51, in purple), and sxcH596F; OgaKO larvae (N = 30, branches: padj = 0.75, branching points: padj = 0.5, in c [file pgen.1010159.s003.tif]

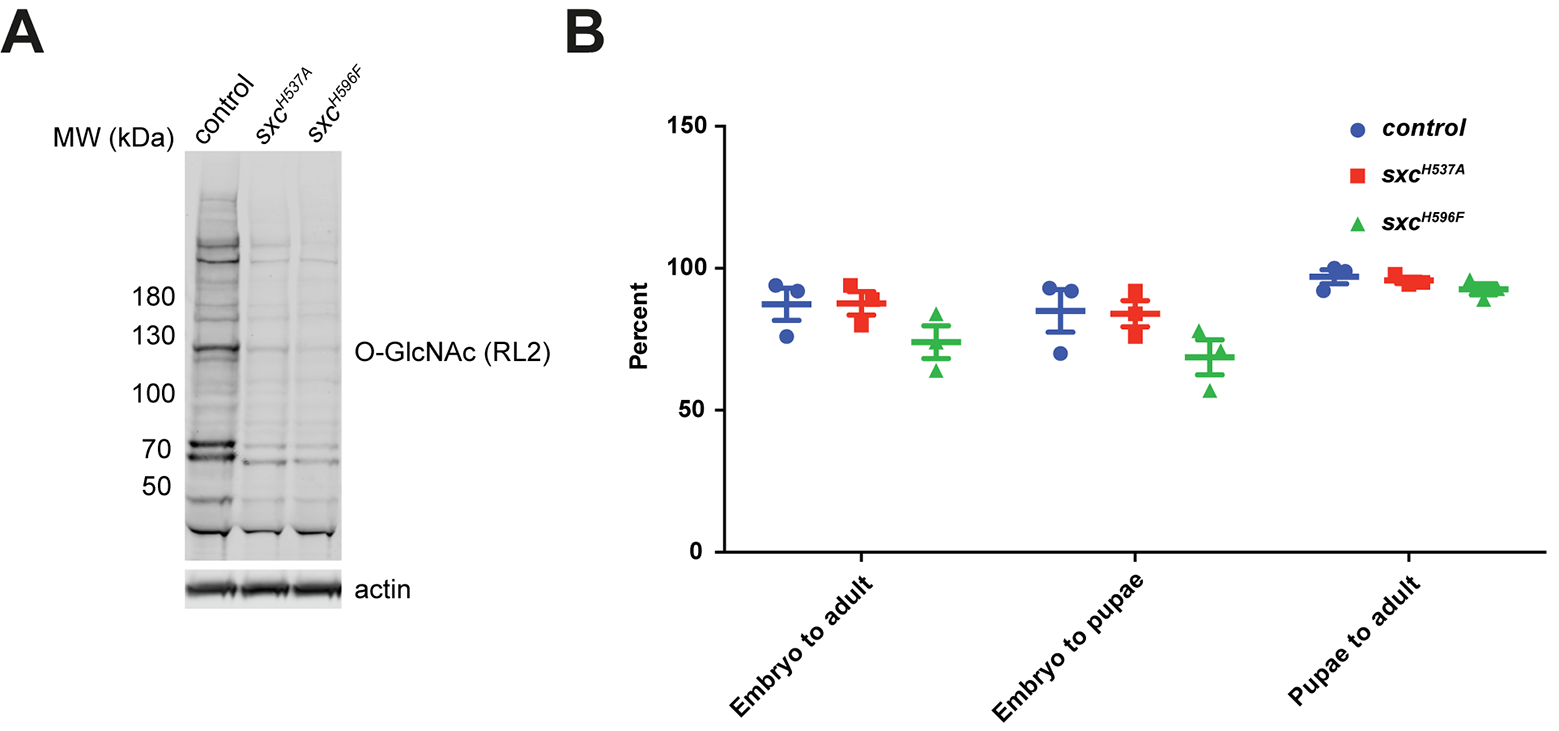

Supplement: S4 Fig — (A) Embryos from either wildtype, sxcH537A, sxcH596F homozygotes were assessed for levels of global O-GlcNAc using a pan-O-GlcNAc antibody RL2. The blot was normalized to actin. This blot is a representative of three experiments. (B) Reduced total O-GlcNAc levels in sxcH596F and sxcH537A homozygotes are not associated with developmental lethality. Data presented as percentage of pupae and adults derived from stage 11–16 embryos (100 per genotype per experiment, n = 3). Based on Student’s t-test with Holm-Sidak’s correction for multiple testing. Complete list of p-values and summary statistics is provided in S3 Table. (TIF) [file pgen.1010159.s004.tif]

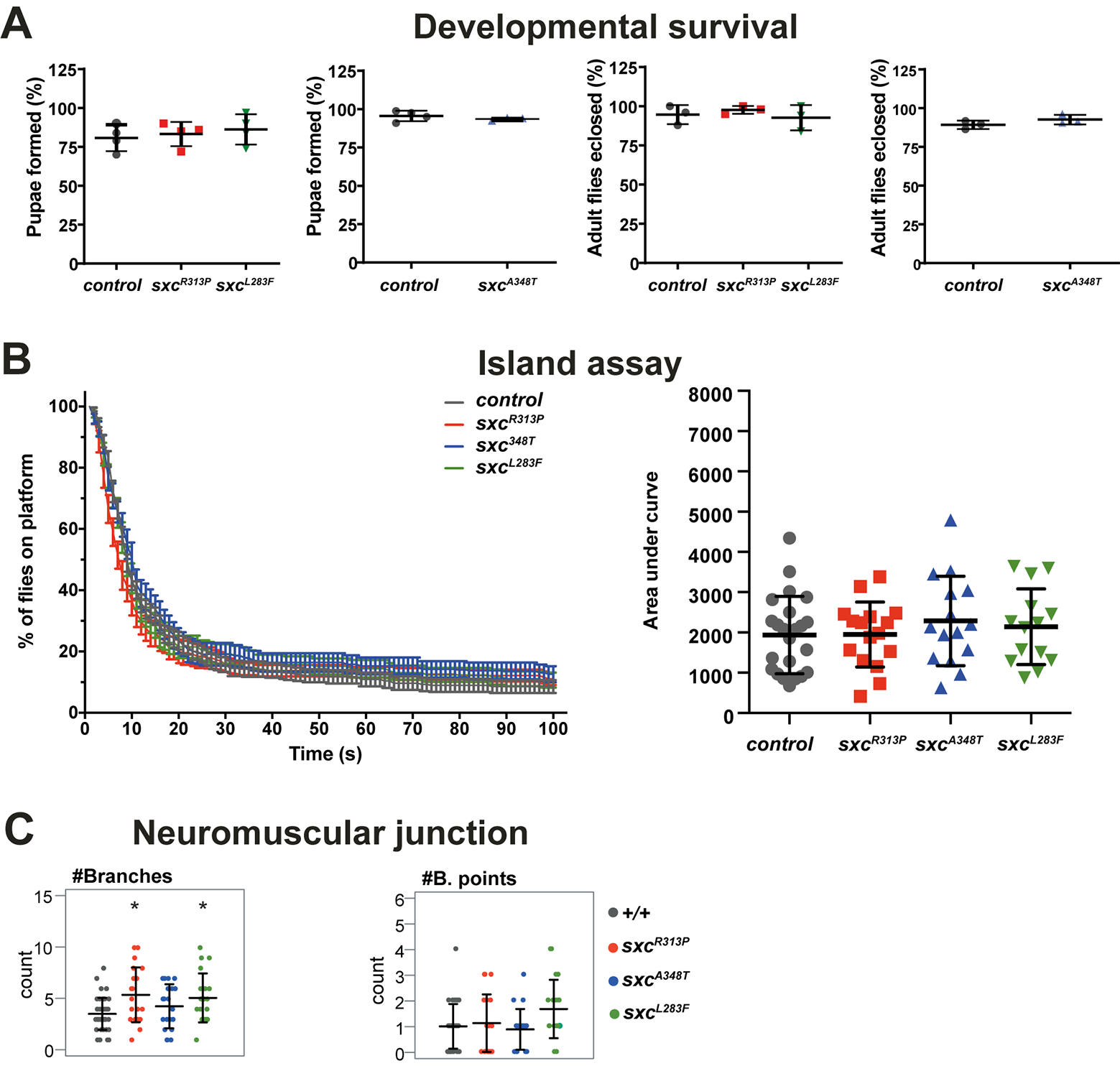

Supplement: S5 Fig — (A) Control, sxcR313P, sxcA348T and sxcL283F embryos (stage 11–16, 80–100 per experiment) were transferred to fresh food at 25°C, and the numbers of pupae formed and adults eclosed were counted. Development from embryo to pupae or from pupae to adulthood was not significantly affected in sxcR313P (pupae: N = 4 repeats, p = 0.9, adults: N = 3, p = 0.29, in red), sxcA348T (pupae: N = 3, p = 0.152, adults: N = 3, p = 0.345, in blue) and sxcL283F mutants (pupae: N = 4, p = 0.108, adults: N = 3, p = 0.727, in green). Data presented as individual data points with mean ± SD. P-values are based on Student’s t-test. (B) Flight escape performance was assessed in the island assay. 15 flies per measurement were thrown on a white platform surrounded with water. Data was collected over 3 days of measurement (Control: N = 23, sxcR313P: N = 16, sxcA348T: N = 15 and sxcL283F: N = 14 repeats). Floating bars depict mean ± SD area under curve (AUC), a parameter that is derived from data plotted as % flies on the platform over time. One-way ANOVA with Tukey’s multiple comparisons was used to compare the mean AUC between genotypes. Flight escape performance of sxcR313P, sxcA348T and sxcL283F flies revealed no defects in locomotion or fitness. (C) Number of synaptic branches is increased in sxcR313P (N = 21, p = 0.049, in red) and sxcL283F larvae (N = 20, in green) compared to the genetic background control (+/+, N = 41, in grey). Number of branching points is not significantly different. Data presented as individual data points with mean ± SD. * p< 0.05. P-values are based on Kruskal-Wallis test with Wilcoxon pairwise test for multiple comparisons. Complete list of p-values and summary statistics is provided in S3 Table. (TIF) [file pgen.1010159.s005.tif]
