# Supplementary material for: Intellectual disability-associated disruption of O-GlcNAc cycling impairs habituation learning in Drosophila
Source: PLoS Genet. 2022 May 2;18(5):e1010159. doi: 10.1371/journal.pgen.1010159 (PMC9140282; doi:10.1371/journal.pgen.1010159)
Supplement: Attachment — Submitted filename: Point_to_point_response_PloSGenet_v7.docx [file pgen.1010159.s010.docx]

Revision of manuscript PGENETICS-D-21-00961, Point by point response to expert referees:

**Reviewer #1:**

The manuscript by Fenckova et al explores cognitive mechanisms in the fly focusing on mutations identified in humans as causative of intellectual disability in the gene O-GlcNAc transferase (OGT). The habituation studies are carefully crafted with well controlled genetic tools. The authors offer important genetic evidence supporting a model where by disruption of the O-GlcNAc addition-removal cycle is causative of cognitive dysfunction in Drosophila models of human mutations. The most clear cut and conclusive evidence is emerging of the Drosophila habituation assays presented in Figures 1 and 4. In my view, these genetic-behavioral experiments provide a solid foundation for the assertion in the title that “Intellectual disability-associated disruption of O-GlcNAcylation impairs […] cognitive function in Drosophila”. However, the paper falls short to provide a comprehensive understanding of processes affected by the diverse mutations tested either individually or collectively. It is clear that global defects on O-GlcNAc-sylation do not account for the habituation phenotypes described in figure 4. Yet, the identity of neural processes and targets are insufficiently explored with a limited data-mining analysis of the Drosophila O-GlcNAc proteome. This last part of the paper is truncated and inconclusive.

While we agree that further in-depth mechanistic studies are of interest and fully warranted, we feel that they are not required to support our messages and therefore fall outside of the scope of this paper. “Providing a comprehensive understanding” of a process and disease mechanism is desired but far from trivial and only rarely achieved by single studies. For instance, decades of research and dozens of high profile papers were required to understand the gross mechanisms underpinning Fragile X Syndrome. The sensitive genetic approach that we successfully used here provides the first evidence for deficits in O-GlcNAcylation as a disease-causing and cognition-relevant mechanism. Our work also identifies blocking O-GlcNAc hydrolysis as a treatment strategy. In the hope to satisfy the referee, we improved the manuscript by incorporating patient-specific *sxc* mutant alleles and characterize their effects on neuronal development. These data strengthen the function of O-GlcNAcylation in neuronal development and provide insight into the effect of patient-specific mutations on synaptic architecture (**Figure 5**, see detailed response below). We also further elaborate on the potential downstream effectors that do not limit to candidates from Drosophila O-GlcNAc proteome (see **Discussion, page 19-20**).

Concerning figures 2 and 3, in my view they need some reconsideration. Figure 3 is a collection of important controls that can go into supplementary materials.

Figure 3 contains developmental survival data, different aspects of locomotor function (island assay (flight), negative geotaxis assay (climbing), and O-GlcNAc western blots. Since the investigated patient-specific mutant alleles did indeed not show defects in developmental survival and island assay, we moved these data to the supplementary material as suggested (now **Figure S5, panels A and B**). We kept the negative geotaxis data that report climbing defect for the *sxc^R13P^* allele and the O-GlcNAc western blot that show unaltered total protein O-GlcNAcylation levels in all three investigated alleles in the main figure. Since there are no space constraints and these data are of significant relevance to our story, we would like to keep these in a main figure. We hope that the referee agrees with our wish.

Figure 2, while I can see the graphs depicting the reported differences in boutons number and other NMJ parameters, I cannot see in the images the phenotypes reported by the graphs. Thus, I cannot concur with the authors that ”Intellectual disability-associated disruption of O-GlcNAcylation impairs neuronal development […]”.This assertion needs better NMJ evidence and/or new approach to document this conclusion.

The reason why the differences between NMJs terminal may not strike the referee, is that they are mostly in the range of 0.8-1.3 fold changes, as shown by the quantitative evaluation. In response to the referee, we worked over the Figure. While we already before attempted to choose representative images, we now made sure that the chosen NMJs represent average values of the quantified parameters, and we provide these in the figure legend. *“The quantitative parameter values of the representative images:* ***(A’)*** *(+/+ | sxcH537A/+): #Boutons (31 | 39), Length (103.7 | 137.8), Area (374.4 | 369.4), Perimeter (245.5 | 306.7)* ***(B’)*** *(elav-Gal4/+ | UAS-sxc/+ | Elav-Gal4>UAS-sxc): #Boutons (45 | 37 | 28), Length (160.0 | 137.5 | 93.3), Area (480.4 | 478.4 | 363.9), Perimeter (407.6 | 290.8 | 243.9)* ***(C’)*** *(+/+ | sxcH537A/+, UAS-sxc/+ | sxcH537A/+; elav-Gal4>UAS-sxc): #Boutons (27 | 34 | 31), Length (107.6 | 144.9 | 114.9), Area (430.6 | 464.7 | 361.7), Perimeter (256.0 | 354.8 | 299.4)* ***(D’)*** *(+/+ | sxcH596F/H596F | OgaKO/KO | sxcH596F/H596F; OgaKO/KO): #Boutons (23 | 43 | 35 | 30), Length (111.5 | 205.6 | 122.9 | 142.5), Area (351.7 | 691.7 | 417.8 | 377.6), Perimeter (245.6 | 544.0 | 274.1 | 318.9).”*

Even if differences are in some cases not obvious with bare eyes (to a readership that does not inspect NMJs routinely, or even to specialists) we find it very important to show representative images, not images that show an extreme phenotype for the sake to make a finding obvious. In the literature, this is unfortunately still done rather frequently. Objective and reliable quantification of differences in this order of magnitude is enabled by the automated macro analysis with standardized settings (published and validated in PMID: 26998933, ***PloS Comput Biol***, 2016 and PMID: 28518121, ***J Vis Exp***, 2017) that was applied on the 3D image stacks, whereby accurate image segmentation is manually validated. Clearly, the bouton phenotype of *sxc^H537A/+^* is mild and although a similar increase is seen in *sxc^H537A/H537A^* (**Figure S3D**) and in *sxc^H537A/+^; UAS-sxc* (**Figure 2C**), it does not reach significance. This is why, to make sure that reduction of OGT catalytic activity is responsible for increased bouton numbers, we in our story followed up and assessed the stronger catalytic hypomorph, *sxc^H596F^*. *sxc^H596^*^F^ mutant larvae showed increased bouton numbers (**Figure 2D**, p = 0.003) as well as other NMJ parameters that were inconsistently affected between homozygous and heterozygous *sxc^H537A^* larvae (length, area and perimeter), validating the NMJ phenotype. In our revised version, we now also analyzed NMJ morphology of the patient-specific *sxc* mutant alleles and found that the *sxc^R313P^*, *sxc^A348T^* and *sxc^L283F^* affect bouton numbers in the same direction as the catalytic mutants (**Figure 5** and new result section on **Pages 13-14, lines 321-337**) “*sxcR313P and sxcA348T and sxcL283F show deficits in synaptic morphology*”). This is in line with the observed habituation deficits that are caused be decreased O-GlcNAcylation. We hope that these adaptations improve the quality of the documentation and convince with our conclusions. If any raw data are desired, be it images, numeric values per synaptic terminal, or both, we will be happy to provide them.

Appendix has references at the end not related to the content of the paper.

Thank you for the feedback! We fixed the problem.

In summary, only two figures provide uncontestable insight (Fig. 1 and 4) making this manuscript a solid yet rather discrete contribution.

We hope that the improved **Figure 2**, additional data on the patient-related *sxc* mutant alleles (**Figure 5**) and explanation why in-depth mechanistic follow-up is not feasible in this manuscript, will satisfy the reviewer’s concerns.

**Reviewer #2**:

My comments and criticisms are minor but I offer those for consideration of the author and editor:
1. I do think that labeling habituation responses as cognitive function will not be well received by most human geneticists. There has always been a tension between model organism researchers and human geneticists regarding when invertebrate models reflect human pathological mechanisms. I would advise not using this term, but simply stating that they have found affects on habituation and synapse development.

We thank the referee for this suggestion and recognize that the use of appropriate terminology is very important. We now refer to habituation as “cognition-relevant”, as supported by a large body of literature, e.g. *Miller et al.,* ***Child Development, 1977***; Gonzalez-Gonzalez et al*.,* ***Acta Obstet Gynecol Scand, 2006****; Kavšek, M.,* ***Journal of Applied Developmental Psychology, 2004****; Rankin, C.H., et al.,* ***Neurobiol Learn Mem****,* ***2009****; McDiarmid et al.,* ***Neurosci Biobehav Rev****,* ***2017***), to only indicate a few. We recently summarized the evidence for the habituation relevance to cognitive function in an invited review article for ***Neurosci Biobehav Rev*** that already is available at DOI: 10.20944/preprints202201.0186.v1.

We also changed the title to: “*Intellectual disability-associated disruption of O-GlcNAcylation impairs neuronal development and cognition-relevant habituation learning in Drosophila*” and the short title to: *“O-GlcNAc cycling controls habituation learning in Drosophila”*.

2. Have they done any CNS morphological assessments of their mutants?

There are many aspects of CNS morphology that could be studied in the *Drosophila* models. The available patient data, however do not indicate a particular aspect of CNS morphology to be affected. Because other studies have already pointed to role of OGT and O-GlcNAcylation in synapse development and maturation, we have focused on synapse development and assessment of neuromuscular junction morphology, were we indeed found phenotypes.

3. I am a bit puzzled by their results with the sxc[H537A] allele. As heterozygotes this allele affects bouton number but not other parameters of synapse size. Yet, when the homozygote is analyzed it displays phenotypes for NMJ length and perimeters but not bouton number. Likewise I noticed that the effect of this allele as a heterozygote on bouton number is a bit modest, and show some variation within experiments shown (for example see the difference between Fig. 2 Panel A, and Figure 2 Panel C. The difference between sxc/+ and +/+ is pretty modest in Panel C, affected largely by a few outliers. I think some further discussion of these findings is warranted. In my mind it tells me the effect of synapse morphology is modest and makes me wonder if there are any observable electrophysiological changes.

We fully agree with the referee and do also not have an explanation for the *sxc[H537A/+]* and *sxc[H537A/H537A]* phenotype discrepancy. The bouton phenotype in the *sxc[H537A/+]* (**Figure 2A**) and especially *sxc[H537A/+];UAS-sxc* mutant (**Figure 2C**) is mild. The observation of the reviewer that the bouton phenotype of the *sxc[H537A/+];UAS-sxc* mutant (**Figure 2C**) is affected by outliers led us to re-inspect all images with extra care. We found a single image in the control dataset of **Figure 2C**, in which presynaptic synaptotagmin staining did not cover the complete NMJ area, possibly due to incomplete penetration of the antibody or damage of the synapse. We excluded this picture from the dataset, updated the graphs in **Figure 2C** and repeated the statistical analysis. After multiple testing correction, the increase in bouton number in *sxc[H537A/+];UAS-sxc* animals is now no longer significant (p = 0.071). What we would like to highlight in the assembled side-by-side panels of all three genotypes below (from figures 2A, 2C and S3D) is that despite the differences in statistical significance, the data is however very consistent. There is a clear quantitatively very consistent tendency of increased bouton number across the three tested H537A mutant conditions; we agree that the phenotype is mild and variable.

We now address this point on **page 9, lines 205-211**: “*Accordingly, we found a significant increase in NMJ length and perimeter in homozygous sxc^H537A^ larvae. The increase in the number of synaptic boutons did not reach significance (****Figure S3D****), potentially due to greater variability. It neither withstood multiple testing correction in the sxc^H537A/+^; UAS-sxc/+ larvae that were used as a control in the rescue experiment (****Figure 2C****). However, there is a quantitatively very consistent increase in bouton number across the three tested H537A mutant conditions (fold changes 1.19, 1.18 and 1.15).* *We therefore conclude that the bouton phenotype associated with H537A mutation is mild*.”

It is because of this discrepancy in statistical significance that we subsequently assessed NMJ morphology of the stronger catalytic hypomorph, *sxc[H596F]*. This mutant showed a more severe phenotype in both bouton and size-related parameters (length, area and perimeter), demonstrating the requirement of sxc in synaptic bouton development. Moreover, in our revised version we now also provide NMJ morphology of the patient-specific *sxc* mutant alleles and found that the *sxc^R313P^*, *sxc^A348T^* and *sxc^L283F^* affect bouton numbers in the same direction as the catalytic mutants (**Figure 5**). This is in line with the observed habituation deficits that are caused be decreased O-GlcNAcylation. We hope that the referee agrees that the improved documentation and additional data fully justify our conclusions.

Secondly, how changes in the NMJ morphology affect physiology is a question that we do not address experimentally, and it is not predictable. Studies in some mutants with increased bouton number indeed found a correlation with increased motorneuron excitability (*Budnik,* ***Curr Opin Neurobiol****, 1996; Schuster et al.,* ***Neuron,*** *1996; Mosca et al.,* ***Proc Natl Acad Sci U S A****, 2005*) but others did not (*Schwenkert et al.,* ***Mech Dev****, 2008*; *Sutcliffe et al.,* ***PloS One****, 2013*; *Schuster et al.,* ***Neuron,*** *1996*). Whereas we would love to be able to generate or see such data, neuronal physiology would not tell us either whether and how do the mutations affect cognitive function. It is for this reason that we directly look at the most cognition-relevant parameter that is known and available to us: habituation.

We now discuss this on **Page 17, lines 433-438:** “*Increased number of synaptic boutons has been also associated with increased excitability at the NMJ [67–69] although not consistently [69–71]. An interesting future direction could involve electrophysiological assessment of NMJ activity to determine whether O-GlcNAc cycling and the patient-related sxc mutations go beyond determining synapse development and affect synapse excitability and/or plasticity. However, these investigations would need to test various aspects of physiology and would still leave the impact of O-GlcNAc on cognition undetermined. For this reason, we assessed habituation as a highly cognition-relevant parameter.”*

4. I found the allele specific analysis very interesting and followed their stepwise arrival at the conclusion that while overall GlycNAcylation was not changed in some alleles, specific protein targets do likely see differences in modification since the phenotypes can be rescued by altering Oga function. However, I am not a big fan of making biological conclusions from multi-step bioinformatics analyses, and while their enrichment analysis seems valid, it is a far cry from doing an experiment. Their ability to look at the patterns of GlycNAcylation by western seems a logical place to begin to see if different alleles give different patterns (ie intensities) of modification. I would be intrigued if this sort of analysis was attempted and if there were any indications of differential modification of protein targets.

We thank the referee for her/his interest. Unfortunately, quantitative O-GlcNAc proteomics analysis is not easily accessible.

We would indeed very much like to follow-up on proposed targets of our enrichment analysis and investigate whether the observed habituation deficits are due to their compromised O-GlcNAcylation. Also further unbiased approaches to identify new targets can be envisaged. Unfortunately either a significant body of additional work thatwe feel is beyond the scope of this manuscript; it requires further funding that we are positive we will be able to raise on the fundament of the present study, which clearly indicates the need for this work

We have modified the text and now emphasize that our analysis is explorative and that requirement of further analysis and experimental validation of the predicted targets is needed. **Page 14, lines 342-345** (the added text is underlined): “*We hypothesized that altered O-GlcNAcylation of specific sxc substrates is responsible for the habituation deficits. We therefore attempted to predict candidate substrates by exploration of the Drosophila O-GlcNAc proteome”…* and **Page 15, line 362-364**: *“Further analysis will be required to answer the question, whether impaired O-GlcNAc activity towards one or more of these targets is responsible for habituation deficits that are associated with OGT-CDG mutations.”*

**Reviewer #3**:

Major points

1) The author performs the habituation assay on a number of sxc mutants but do not show data for the null allele. I acknowledge the homozygous null flies are pupal lethal but the authors should be able to test this in a heterozygous fly. Since the null allele is an important reference point to interpret the function of missense alleles (e.g. if the mutant phenotype of certain missense allele is stronger than the null, they maybe antimorphic or neomorphic alleles, rather than a hypomorph), it would be valuable to show what the null allele looks like in this assay (which have been generated by the authors in PMID: 29588363. In addition, multiple alleles are available from stock centers). Also, note that the habituation data for the strong hypomorphic allele (H596F) they generated is also not provided here, which would make this paper more complete.

As the referee correctly noted, homozygous null animals are not viable and for this reason cannot be tested. We indeed had not measured the *H596F* allele, as we expected at least the homozygous *sxc[H596F/H596F]* flies to be too affected to absolve the assay. We thank the reviewer for the proposal to test heterozygous flies, with the goal to establish the phenotype resulting from a validated loss of function condition.. Upon the referee’s request, we now attempted to assess habituation of *sxc[H596F/+],* but also *sxc[H596F/H596F]* catalytic hypomorph flies. We found the jump performance as illustrated by the initial jump performance score of both heterozygous and homozygous mutants to be compromised to a degree precluding determining habituation. We report this phenotype on **page 10, lines 221-224**: “*We also subjected flies carrying the sxc^H596F^ allele to the habituation assay, but both homozygous as well as heterozygous sxc^H596F^ flies showed an impaired jump response (38% and 42% initial jumpers), similar to homozygous sxc^H537A^ and pan-neuronal sxc knockdown flies. This precludes assessment of habituation*.”

Of note, we do not expect other mechanisms than (partial) loss of OGT catalytic activity to be involved in their habituation phenotypes because the habituation deficits of *sxc[R313P/+]* and *sxc[A348T/A348T]* flies are fully restored by blocking O-GlcNAc hydrolysis. This is consistent with a ~2-fold decrease in catalytic efficiency and impaired glycosylation kinetics that was shown in purified recombinant TPR domains carrying the equivalent OGT-CDG mutations (R284P, A319T, L254F) (Selvan et al., 2018, **J Biol Chem**). Molecular dynamics simulations derived from crystal-based structure of OGT-TPR domain further revealed that these mutations significantly alter substrate binding properties (Llabres et al., 2019, **J Struct Biol**). We refer to these findings in the Introduction. **Page 5, lines 83-92** (the added text is underlined)**: “***The OGT protein consists of an N-terminal tetratricopeptide (TPR) domain, which contributes to substrate recognition and binding [35], and a C-terminal catalytic domain that is responsible for glycosylation of the TRP-bound proteins. OGT-CDG mutations have been found in both domains. Unlike the mutations in the catalytic domain, the mutations in the TPR domain have not been shown to significantly affect global protein O-GlcNAc levels but they do affect the OGT-TPR domain substrate binding and glycosylation kinetics as derived from in vitro assays and crystal structure analysis [26,35,36]. However, it remains to be known whether impaired glycosylation is the mechanism that leads to developmental and cognitive defects caused by the TPR domain mutations. This question is pertinent as the TPR domain has been shown to be essential for cellular functions other than glycosylation [38].”* and in the Discussion section. **Page 18, lines 459-462**: “*All OGT-CDG mutations investigated in this study were shown to impair the substrate interaction properties and the glycosyltransferase kinetics [27,36]. The observed habituation deficits may thus be caused by impaired O-GlcNAcylation dynamics towards a specific set of substrates that cannot be captured by standard O-GlcNAc detection assays.”*

We agree with the referee that examination of the (heterozygous) sxc null mutant would make our study yet more complete, but since the weaker *sxc^H596F^* catalytic hypomorph already affects the light-off jump response and precludes habituation assessment, it is conceivable/most likely this will be the case also for any catalytically inactive (eg. *sxc^K872M^* that we published in PMID: 29588363) and null heterozygous *sxc* mutant. They represent stronger alleles with zero catalytic activity.

We have examined the NMJ morphology of the OGT-CDG mutants, as the referee suggested, and found that this resembles that of the catalytic hypomorphs (see in response to comment 2), thus not directly pointing to other mechanisms. We feel, also in light of the previously published data that point to negative effect on OGT catalytic activity as the underlying disease-causative mechanism, that examination of the *sxc* null allele has little additive value.

2) The authors explored the synaptic morphology phenotype of the known enzymatic defective mutants (H537A and H596F), but have not examined this for the patient derived variants (R313P, A348T, L283F). Was this because they looked at these phenotypes and did not see any relevant phenotype? Or was there a reason this experiment cannot be carried out? Since the authors are trying to make the point that these synaptic phenotype has some relevance to human ID, it would make sense to show how the NMJs of the flies with mutations that are analogous to those found in the patient mutations look like. Also, data from the heterozygous or homozygous null allele can also be provided here to strengthen this paper.

In this study we focused our analysis of the OGT-CDG mutations in cognition-relevant readout – habituation, and less in NMJ morphology. The reviewer is right that the synaptic phenotypes of these mutant alleles could have been assessed. If affected the Drosophila NMJ model could then be utilized for investigation of the underlying mechanisms. Following the referee’s suggestion, we now analyzed and provide NMJ morphology of the three OGT-CDG homozygous patient-relevant alleles. The results and representative images can be found in **Figure 5** (boutons, length, area, perimeter) and **Figure S5** (branches, branching points). All three mutant alleles display significant increase in number of synaptic boutons and *sxc^R313P^* and *sxc^L283F^* show also increase in other size-related parameters, length (*sxc^R313P^*), perimeter (s*xc^R313P^*) and number of synaptic branches (*sxc^R313P^* and *sxc^L283F^*). Thus, the patient-relevant mutations affect NMJ parameters in a similar manner as the catalytic hypomorph mutations. We added a paragraph with complete result description and conclusion to the manuscript text: “*sxc^R313P^ and sxc^A348T^ and sxc^L283F^ show deficits in synaptic morphology*” (**page 13-14**).

3) The authors attribute the negative geotaxis defect of the R313P allele to this variant affecting a non-enzymatic function of Sxc. While this is an interesting idea, alternatively this phenotype may be due to some sort of a 2nd site mutation that is unique to this strain (off targeting by CRISPR or some floating mutation that is closely linked that was not eliminated through back crossing). The authors should perform a rescue experiment (e.g. neuronal rescue experiment using UAS/GAL4 as they do for other alleles or genomic rescue) if they wish to claim that the negative geotaxsis defect in this allele is due to sxc and not other genes.

We thank the referee for raising this point. We backcrossed all mutant alleles to the control genetic background for six generations to eliminate the effect of possible second site hits. We can however not formally exclude that the negative geotaxis defect in the *sxc^R313P^* line is caused by a secondary mutation located in the close vicinity of the *sxc* gene that was not eliminated by backcrossing. We indeed considered a genetic rescue by mutant *Oga,* although both *Oga^KO^* and *Oga^D133N^* mutations are associated with climbing defects. This experiment would tell us whether the climbing defect is or is not caused by reduced O-GlcNAc transferase activity. We know that it is not because catalytic mutants do not have climbing defects. We tested them for this reason. An effect of off-target/second site mutation thus still cannot be excluded by this approach. We also took distance from a genetic rescue by overexpressing *sxc* cDNA using a UAS/GAL4 approach in the *sxc^R313P^* background, based on our experience that with highest likelihood, this would lead to OGT activity far exceeding the wild-type level, with no decrease in global O-GlcNAcylation. We now acknowledge the possible effect of an off-target mutation on the negative geotaxis phenotype and adapted our conclusion. **Page 11, lines 265-267**: “*This effect appears to be independent of the O-GlcNAc transferase activity. However, a contribution of a potential second site mutation affecting another gene that was not eliminated by six generations of backcrossing cannot be formally excluded*.”

4) The section subtitle “Patient-related sxc mutant alleles have normal levels of O-GlcNAcylation” seems a bit misleading, especially since they later argue that phenotypes seen in some of the patient mutant alleles can be corrected by removing one copy of Oga. As they discuss in the text that there may be some minor O-GlcNAcylatiojn defect that is not obvious when performing a bulk O-GlcNAcylation assay by western blot (e.g. a few substrates that are more sensitive to slight reduction in O-GlcNAcylation is responsible for the habituation and synaptic phenotypes). The authors may want to consider an alternative title like “Patient-related sxc mutant alleles do not affect global protein O-GlcNAcylation”.

We thank the referee for pointing this out. We changed the heading of this section to “*Patient-related sxc mutant alleles do not affect global protein O-GlcNAcylation*”, as suggested.

Minor Points:

1) This last sentence of the abstract (“This study establishes a critical role for O-GlcNAc cycling and disrupted O-GlcNAc transferase activity in cognitive dysfunction and intellectual disability and points to potential treatment strategy for OGT-CDG.”) is a bit length with many “and” being used. Better to split into two sentences or rephrase.

We adapted the last sentence of the abstract and we split the sentence for better readability. It now reads: *“This study establishes a critical role for O-GlcNAc cycling and disrupted O-GlcNAc transferase activity in cognitive dysfunction. It also reveals blocking O-GlcNAc hydrolysis as a potential treatment strategy for OGT-CDG.”*

2) The fly ortholog of OGT (sxc) has been reported to be a member of the Polycomb group as the authors discuss. Considering that many members of Polycomb genes are associated with human diseases that affect the nervous system including ID (reviewed in papers such as PMID: 34426021), the author may want to discuss the potential connection between OGT, Polycomb genes and O-GlcNAcylation, if there may be a link.

We thank the referee for this input. We added to our discussion (**page 19-20, lines 479-494**):

“*Drosophila sxc is a member of polycomb group (PcG), a conserved set of chromatin and transcriptional modifiers that is required for maintenance of transcriptional repression (of non-lineage genes) during embryonic development and cell proliferation [references 79,80]. Missing O-GlcNAcylation of PcG component Polyhomeotic (Ph) is responsible for misexpression of HOX genes and homeotic transformations in sxc null mutants [reference 81]. A recent study has shown that chromatin redistribution induced by interaction between sxc and PcG member Polycomb like (Pcl) controls plasticity of sensory taste neurons [reference 82]. It is not known whether the regulation of PcG activity by sxc/OGT is important for cognitive function but it can be noted that a series of PcG genes are associated with ID [references 83,84], and some of them are subjects of OGT regulation in developmental or cancer context. These include PHC1 – human ortholog of Drosophila Ph [reference 85], RING1B (Drosophila Sce) [references 86,87], EZH2 (Drosophila E(z)) [references 88–90], YY1 (Drosophila pho) [references 91,92] and ASXL1 (Drosophila Asx) [references 93,94]. In addition, OGT regulates expression of PcG genes by O-GlcNAcylation of PcG transcriptional regulators, for example ATN1 (Drosophila Gug), which was identified in the embryonic O-GlcNAc proteome [references 59]. The encoded proteins represent interesting candidate targets that may link cognitive deficits of OGT-CDG mutations to PcG function.”*

To accommodate this paragraph, we established a new subsection in the discussion: “Potential downstream effectors of O-GlcNAcylation in cognition and cognition-relevant processes”. It now unifies all our discussion points on the potential downstream mechanisms.

3) There is one prior study that found a role for OGT in the nervous system, specifically in the context of circadian rhythm regulation using timeless-GAL4 and UAS-RNAi. Although this study may not be directly relevant to this work, the author may want to consider citing this it as one previous evidence in flies that this gene has critical functions in the nervous system (note that this reviewer is not the author of this study nor have any conflict of interest).

We thank the referee for pointing our attention to this study. We now cite it in the Introduction (**page 5, lines 99-100**).

**Reviewer #4**:

Major Revisions:

1. In both the title and throughout the Introduction and Discussion, the authors use the term ‘cognitive function’ lightly, basing this on the habituation defects observed in their mutants. However, cognition generally describes more complex, executive functioning and its use may misrepresent the experimental findings. A more fitting word choice would simply be ‘habituation’.

We thank the referee for this suggestion and recognize that the use of appropriate terminology is very important. We now refer to habituation as “cognition-relevant”, which is supported by literature (*Miller et al.,* ***Child Development, 1977***; Gonzalez-Gonzalez et al*.,* ***Acta Obstet Gynecol Scand, 2006****; Kavšek, M.,* ***Journal of Applied Developmental Psychology, 2004****; Rankin, C.H., et al.,* ***Neurobiol Learn Mem****,* ***2009****; McDiarmid et al.,* ***Neurosci Biobehav Rev****,* ***2017***). We recently summarized the evidence for the habituation relevance to cognitive function in an invited review article for ***Neurosci Biobehav Rev*** that is available at DOI: 10.20944/preprints202201.0186.v1.

We also changed the title to: “*Intellectual disability-associated disruption of O-GlcNAcylation impairs neuronal development and cognition-relevant habituation learning in Drosophila*” and the short title to: *“O-GlcNAc cycling controls habituation learning in Drosophila”*.

2. In the learning and memory literature, habituation as a form of learning is accepted once a number of criteria are met. First, the decreased response should not be fatigue (which the authors effectively rule out) and sensory adaptation should not explain the results (this is unaddressed by the authors). To suggest learning, the habituated response should also show a) recovery by dishabituating stimuli (an air puff, for example), b) that the jump response recovers spontaneously in a stimulus-dependent fashion, and c) that the habituation is stimulus specific. The authors should address (experimentally or through literature review) at least one of these criteria (a-c) before claiming that learning is being measured. If they or others have shown that sensory adaptation does not explain the habitutation, that too should be documented.

We agree with the referee that the paradigm used to study habituation must fulfill the defined characteristics. We want to assure the referee that the light-off jump habituation that we use in this study fulfills 8 out of 9 criteria for short-term habituation (defined by Thomson, **Psychol Rev**, 1996 and revisited in Rankin et al., **Neurobiol Learn Mem**, 2009). Most importantly: a) dishabituation (with air puff dishabituating stimulus) and b) spontaneous recovery were demonstrated by Engel and Wu, 1996, **J Neurosci**). They have also shown that habituation of the jump response can be induced by stimulation downstream of the sensory areas, excluding c) sensory adaptation.

We further documented in our own work and experimental setup that the flies jump specifically to the light-off stimulus and not to a dimmed light = weaker stimulus, excluding sensory hypersensitivity (Fenckova e al., **Biol Psy**, 2019, **Figure S3B**). The jump response also occurs immediately after the light-off stimulus (within the first 100 ms) in each light-off trial and the flies do not jump repeatedly between the trials (Fenckova e al., **Biol Psy**, 2019, **Figure S3A**). Thus, any form of behavior hyperexcitability should not confound the observed habituation deficits.

We now provide this information in the text and refer to the original publications. **Page 6-7, line 134-138** (added text underlined): “*In habituation, an initial strong response towards a repeated but harmless stimulus gradually wanes based on prior experience. To assess this phenotype we used light-off jump habituation, an established non-associative learning assay that meets the strict habituation criteria, including spontaneous recovery and dishabituation with novel stimulus and excluding sensory adaptation and motor fatigue [46,52].”*

3. While the Introduction was informative as to the importance of O-GlcNAcylation, the authors make frequent interpretations of their habituation assay and of sxc function in the Results that are only explained after reading the Discussion, and then only briefly. In particular, the reader would benefit from a short introduction to a) habituation learning, b) the function of the TPR domain of sxc in relation to the glycosyl transferase catalytic region, and c) a structure-function analysis of ID-specific mutations (through crystallography), and their known effects (if any) on substrate interactions.

We thank the referee for this important point that had escaped our attention. We included an introduction to habituation learning and also to the synapse morphology assay (because this indeed was also introduced only later at the Result section) in the Introduction section. The text now reads as (**page 5-6, lines 102-113**, added text underlined)…”*Here we genetically manipulated both O-GlcNAc transferase and O-GlcNAc hydrolase activity using established sxc and Oga mutants and investigated the effect of O-GlcNAc cycling on cognitive function and neuronal development. “We turned to habituation and synaptic morphology, two parameters relevant to neuronal function and development. Habituation is an evolutionary conserved form of non-associative learning that is characterized by response decrement towards a repeated, non-meaningful stimulus. At the neuronal level, habituation is mediated by adaptive changes in the excitatory activity of the stimulus response pathway causing attenuated downstream neuronal responses to repeated, familiar stimuli. Habituation serves as a filter mechanism that prevents information overload and allows the cognitive resources to focus on relevant matters [43]. It represents a prerequisite for higher cognitive functions [41–46]. Deficits in habituation have been reported in a number of neurodevelopmental disorders [47] and in more than a hundred Drosophila ID models 48,49]. Neuronal development was investigated with the assessment of synaptic morphology at the Drosophila neuromuscular junction, an established model synapse [50].”*

We also added more information about the TPR domain and its function in relation to the catalytic domain to the Introduction. We describe what is known about the effect of the TPR domain mutations for the glycosylation kinetics. **Page 5, lines 83-92** (added text is underlined): **“***The OGT protein consists of an N-terminal tetratricopeptide (TPR) domain, which contributes to substrate recognition and binding [35], and a C-terminal catalytic domain that is responsible for glycosylation of the TRP-bound proteins. OGT-CDG mutations have been found in both domains. Unlike the mutations in the catalytic domain, the mutations in the TPR domain have not been shown to significantly affect global protein O-GlcNAc levels but they do affect the OGT-TPR domain substrate binding and glycosylation kinetics as derived from in vitro assays and crystal structure analysis [26,35,36]. However, it remains to be known whether impaired glycosylation is the mechanism that leads to developmental and cognitive defects caused by the TPR domain mutations. This question is pertinent as the TPR domain has been shown to be essential for cellular functions other than glycosylation [38].”*

4. Regarding the light-off jump habituation, the data indicates that not only do genetic manipulations of O-GlcNAcylation alter the rate of habituation, but they also influence the post-habituation baseline activity level (indicated by higher baselines in Figure 1 and Figure 4). Furthermore, the rescue of the trials to criterion (TTC) throughout the paper correlates with the return of this baseline activity back to control levels. The authors should explain their interpretation of this higher baseline at its first occurrence in the Results.

The average jump response curves in Figure 1 and 4 depict the % of jumping flies in each light-off trial (described in the figure legends). The higher baseline is caused by mutant flies habituating later, receiving a higher TTC score (Trials To no-jump Criterion). In the extreme case, individual flies do not habituate at all and receive TTC score of 100. Controls contain very few or none of these non- or late-habituators leading to low TTC scores. Thus, TTC scores and baseline levels grossly correlate (low TTC > low baseline level, high TTC > high baseline level). The only possible alternative scenario that could interfere with this correlation would be if mutants quickly habituate or stop to jump for other reasons (fatigue, which we have excluded), but then re-start jumping later again. Such a scenario would lead to a low TTC but high baseline. We have never observed a high TTC and low baseline, and find it challenging to think of a possible biological underpinning. In our previous studies on a large number of genotypes, we typically see, as expected, the correlation between TTC and baseline (see e.g. Fenckova, **Biol Psy**, 2019 (<https://doi.org/10.1016/j.biopsych.2019.04.029>; **Figure 1B,C,D**); van Bon, **Hum Mol Genet**, 2013; Willemsen, **J Med Genet**, 2013; Lugtenberg, **Eur J Hum Genet**, 2016; Esmaeeli-Nieh, **Plos Genet**, 2016; Stessman, **Am J Hum Genet**, 2016; Stessman, **Nat Genet**, 2017). We have attempted to improve our explanation of the habituation deficit in the text (**page 7, lines 144-148**): “*Compared to control flies that habituated quickly to the repeated light-off stimulus, sxc^H537A/+^ flies displayed significantly slower habituation and needed significantly more light-off pulse trials to suppress their jump response (Trials To no-jump Criterion, TTC). Some mutant flies were not able to suppress their jump response during the entire course of the experiment, as reflected by the high baseline of the average jump response curve (****Figure 1A****).”*

I am also wondering if, since the authors are measuring wing vibration and the actual jump frequency, there may be a change in the animal’s excitability. Given the change in NMJ bouton number (increased in some genotypes), a change in neuronal excitability might also be hypothesized in the Discussion.'

Yes, a defect in habituation – the failure to plastically adapt/reduce the excitatory jump circuit in response to repeated stimuli - per definition includes an altered excitability in the advanced phase of the experiment (see introduction on neuronal mechanisms underlying habituation that we added to **page 5, lines 106-108**): ”*At the neuronal level, habituation is mediated by adaptive changes in the excitatory activity of the stimulus response pathway, causing attenuated downstream neuronal responses to repeated, familiar stimuli.*” The referee expresses the interesting idea that increased neuronal excitability may be associated with increased synaptic bouton numbers and may underlie habituation deficits of the *sxc* mutant flies. We highly appreciate the efforts of referee to help us to mechanistically link our phenotypes. There is the possibility that both are linked, but we feel that to discuss this is highly speculative and could come across as an overstatement. First, we do look at the larval NMJ versus adult behavior, which per see excludes that the observed (larval) structural defects are directly related to the (adult) behavior. Second, increased transmission/excitation could originate from any other level of the jump circuit than motorneurons. Third, the relation between NMJ morphology and transmission is not predictable, depends on the gene. Some mutants with enhanced neuronal activity indeed show an increase in bouton number but others not (see response to Reviewer 2, point 3). For all of these reasons, we would prefer to keep any discussion in this direction very short. We have added the following discussion to the manuscript (**Page 17, lines 433-438**)**:** “*Increased number of synaptic boutons has been also associated with increased excitability at the NMJ [67–69] although not consistently [69–71]. An interesting future direction could involve electrophysiological assessment of NMJ activity to determine whether O-GlcNAc cycling and the patient-related sxc mutations go beyond determining synapse development and affect synapse excitability and/or plasticity. However, these investigations would need to test various aspects of physiology and would still leave the impact of O-GlcNAc on cognition undetermined. For this reason, we assessed habituation as a highly cognition-relevant parameter.”*

Minor Revisions:

1. Throughout the text there numerous grammatical errors (missing ‘the’, ‘for’, ‘a’, for example).

We have carefully checked the text and corrected the grammatical errors

2. On page 6, lines 123 and 124, and then again on page 7, lines 148 and 149, the authors present data without showing the n or any statistics. These additions will strengthen the author’s conclusions and should be included.

We now included the number of flies tested for initial jump response into the brackets. We do not apply any statistics to initial jump response. We use a previously established threshold of 50% flies (of all tested flies in the three experimental repeats) that jump in at least one of the first five light-off trials that is required for assessment of habituation. This is explained and supported by the above mentioned reference on **page 7, lines 141-144**).

3. The first sentence of the paragraph at the bottom of page 12 (lines 288-291) is a run-on and should be broken into two.

We fully agree. The text (now on **page 14, lines 341-344**) reads: “*ID-associated mutations in sxc do not globally reduce protein O-GlcNAcylation yet blocking O-GlcNAc hydrolysis can completely restore the learning deficits in light-off jump habituation. We hypothesized that altered O-GlcNAcylation of specific sxc substrates is responsible for the habituation deficits.*”

4. At the end of page 15 and the top of page 16 of the Discussion the authors discuss the link between O-GlycNAcylation and microtubule polymerization, but they don’t indicate the details that may be relevant – mechanisms of the modulation, for example, which may be relevant to the NMJ length measurements.

We discuss the involvement of microtubule polymerization because a similar mechanism underlies increased synaptic length in Fmr1 and VAP-33A mutants. We now improved this section, explaining the mechanisms, and how they relate to the observed length phenotypes (**page 17, lines 419-431**): “*NMJ size and the number of synaptic boutons in our model is determined by the level of sxc activity. Dependence of these parameters on gene activity/dosage was previously established in Fmr1 (the Drosophila model of Fragile X Syndrome) [59] and other Drosophila models of neurodevelopmental or neurological disorders, including Prosap/SHANK mutants (modelling Phelan-McDermid Syndrome caused by mutations in SHANK3, characterized by ID and ASD), Neuroligin 4 (ID and ASD caused by mutations in NLGN4), VAP33 (model of Amyotrophic Lateral Sclerosis caused by mutations in VAP-33A) and highwire (potential therapeutical target in traumatic brain injury) [63–66]. The synaptic phenotypes associated with impaired sxc catalytic may be linked to increased microtubule polymerization, since it has been shown that O-GlcNAcylation of tubulin negatively regulates microtubule polymerization and neurite outgrowth in mammalian cell lines [70] and Fmr1 and VAP-33A control synaptic growth and bouton expansion through presynaptic organization of microtubules [59,66].”*

5. In Figure 2 A’-D’, larval NMJ synapses are labeled with a pre- and post-synaptic marker. There are also arrowheads to distinguish the type 1b synapses from other synapses. However, there appear only type 1b boutons in the images, making the arrowheads unnecessary.

We carefully rechecked the images and kept the arrowheads only where other synapses (mostly type 1s) are visible. This also applies to the images that we replaced in response in response to reviewer #1.

We greatly appreciated the time that the referees dedicated to help us improving our manuscript. We undertook significant efforts to adjust the manuscript and hope we accordingly and respond in detail, and believe we have addressed all concerns satisfactory. We now acknowledge the unknown expert referees for their help to improve our manuscript.
