# Supplementary material for: Intellectual disability-associated disruption of O-GlcNAc cycling impairs habituation learning in Drosophila
Source: PLoS Genet. 2022 May 2;18(5):e1010159. doi: 10.1371/journal.pgen.1010159 (PMC9140282; doi:10.1371/journal.pgen.1010159)
Supplement: Attachment — Submitted filename: Re-review of Fenchova et al.docx [file pgen.1010159.s011.docx]

**Manuscript #: PGENETICS-D21-00961**

**New Title:** Intellectual disability-associated disruption of O-GlcNAcylation impairs neuronal development and cognition-relevant habituation learning in *Drosophila*

**Summary of author revisions:** Fenckova et al. have thoroughly revised and, in my opinion, strengthened this manuscript. Their attention to detail with regard to scientific writing (and the addition of new data) add clarity to their presentation and provide deeper scientific insight into potential mechanisms. I feel that my major revision points have been adequately addressed. The authors’ changes to the title reduce the perception of ‘over-selling’ their data, the authors now explain important details and provide experimental observations regarding the habituation assay, they now better introduce all of the aspects of their experimental work in the Introduction, and they begin to address the potential for excitability changes in their mutant flies within an expanded Discussion. The authors also adequately address all of my minor grammatical and mechanistic revision suggestions. Furthermore, I appreciate the way in which the authors address the points brought up by each of the other three reviewers. I now feel more confident in suggesting this paper for publication in PLOS Genetics.
